# Supplementary material for: Infectious Risk in Pediatric Emergency Departments in Italy: A Survey by the Italian Society for Pediatric Emergency and Urgent Medicine (SIMEUP) on Available Preventive and Diagnostic Tools
Source: J Clin Med. 2024 Dec 19;13(24):7762. doi: 10.3390/jcm13247762 (PMC11727668; doi:10.3390/jcm13247762)
Supplement: Supplementary file 1 [file jcm-13-07762-s001.zip › jcm-3212756-supplementary.pdf]

Supplementary Material S1

Survey on the Infectious Risk by the Italian Society for Pediatric Emergency and Urgent Medicine (SIMEUP)

AGE
25-35 years old □
35-45 years old □
> 45 years old □

REGION

TOWN

YOUR HOSPITAL IS:
Hospital with < 24 hours pediatric assistance □
Hospital with 24 hours pediatric assistance □
Hospital with I level Emergency Department □
Hospital with II level Emergency Department □
Health care hospital □
Pediatric health care hospital □
Scientific hospitalization and treatment institutions □
Other □

OPERATIVE UNIT

WORK ROLE
Medical doctor □
Resident □
Nurse □
Other □

NUMBER OF PEDIATRIC ACCESSES IN YOUR HOSPITAL
< 10.000 □
10000-20000 □
20000-30000□
> 30000 □

IF YOUR HOSPITAL IS NOT A PEDIATRIC HEALTH CARE HOSPITAL:
Is there a specific access for children in general Emergency care unit? YES NO
Is there a specific path for children to reach fast track or emergency care unit? YES NO

IN THE WAITING ROOM:
- are masks available? YES NO
- are gel and cleaning papers available or is it possible to wash hand? YES NO

- is adequate social distancing guaranteed? YES NO
- is information material on infectious disease trasmission available? YES NO
- is information material available also in foreign language? YES NO

IN THE VISIT ROOM:

-is there a sink? YES NO

- during visit, workers wear adequate protection devices? YES NO

-did workers receive adequate training for use of protection devices? YES NO

-are parents informed of infection risk? YES NO

IS A SPECIFIC INFECTIOUS TRIAGE IN YOUR HOSPITAL? YES NO

ROOMS FOR SHORT-STAY OBSERVATION:

-are single? YES NO

-have a bathroom? YES NO

-are infectious children separated from other patients? YES NO

IN YOUR EMERGENCY DEPARTMENT

-is there a filter zone to wear and undress protection devices? YES NO

-are there disinfection protocols? YES NO

-how many times are surfaces disinfected? YES NO

-if toys are available, how many times they are sanitized? YES NO

-is there an air recicling available? (6 times / hours) YES NO

-has the entrance door an automatic opening or has a handle? YES NO

IN YOUR EMERGENCY DEPARTMENT ARE THE FOLLOWING RAPID TEST AVAILABLE?

-Urine stick □ Model

-Rapid blood count □

-Procalcitonin

-quantity □

-qualitative □

-semiquantitative □

-Rapid reactive C protein □

- Capillar blood gas analysis □

-Rapid pharyngeal swab for S. pyogenes □

-Rapid nasal swab for Respiratory Syncytial Virus □

-Rapid nasopharyngeal swab for Adenovirus□

-Rapid test for fecal antigen

Rotavirus □

Adenovirus □

Norovirus □

Rapid test for IgM against

Chlamydia pneumoniae □

Mycoplasma pneumoniae □

- Rapid antigenic test for SarsCov2 □

- Rapid combined swab for flu □
- Other □

WHERE ARE RAPID TESTS PERFORMED?
-Specific room □
- Visit room □
-Lab
 24 hours □
 Only in specific hours □
-Other

WHO DOES PERFORM RAPID TESTS?
-Nurse □
-Doctor □
-Other □

WHO DOES INTERPRET RAPID TESTS?
-Nurse □
-Doctor □
-Other □

IN YOUR OPINION, RAPID TESTS ARE DETERMINAT FOR DIAGNOSIS AND THERAPY?
-yes □ Specify what kind of test
-no □
-maybe □
- other □

IN YOUR OPINION, RAPID TESTS CAN AVOID UNNECESSARY ANTIBIOTIC THERAPY?
-yes □ Specify what kind of test
-no □
-maybe □
- other □
